# Tricuspid valve calcification in familial pulmonary alveolar microlithiasis: A case report

**DOI:** 10.1016/j.amsu.2020.05.039

**Published:** 2020-05-30

**Authors:** Shaher Samrah, Hanouf Shraideh, Sukiana Rawashdeh, Basheer Khassawneh

**Affiliations:** a Department of Medicine, Faculty of Medicine, Jordan University of Science and Technology, Irbid, Jordan; b Resident, King Abdullah University Hospital, Jordan

**Keywords:** Pulmonary alveolar microlithiasis, SLC34A2, Pulmonary calcifications, Tricuspid calcification

## Abstract

**Background:**

Pulmonary Alveolar Microlithiasis (PAM) is an uncommon, gradually progressive and eventually fatal hereditary disease that affects young population. Familial cases account for up to 50% of reported cases. There are few described cases of extrapulmonary manifestations of PAM and rare reports of cardiac involvement.

**Case report:**

A 45-year-old male patient presented to our center with progressive shortness of breath and dry cough. On physical examination, he was tachypneic and chest examination revealed diminished breath sounds with bilateral early inspiratory crackles. Further workup revealed the diagnosis of PAM. Echocardiography revealed calcifications covering the tricuspid valve with elevated right ventricular systolic pressure. He reported having two sisters with similar illnesses and chest radiographic abnormalities, one died at the age of 38 years from respiratory failure and the other is 42-year-old and still alive and was diagnosed with PAM. Another 35 member of his family were diagnosed with PAM. Unfortunately, few days after discharge, he arrested at home.

**Conclusions:**

Recently, type-II sodium/phosphate co-transporter has been identified in a human aortic valve. Studies have suggested penetrance of mutations of SLC34A2 gene might explain such variability of pulmonary and extrapulmonary involvement. Our case reports a familial cluster of PAM, and the first case of concomitant tricuspid calcification. This finding might be a useful in the investigation for a future genetic targeted therapy.

## Highlights

• PAM is a fatal hereditary disease that affects young population.
• There are few described cases of extrapulmonary manifestations of PAM.
• Mutations of SLC34A2 gene might explain variability of extrapulmonary involvement.

## 1 Introduction

Pulmonary Alveolar Microlithiasis (PAM) is a rare pulmonary disease in which there is deposition of calcium phosphate microliths within the alveoli [1]. PAM is prevalent among family units with high rates of consanguinity among parents of affected individuals. Mutations in the SCL34A2 gene that encodes for a sodium-phosphate type-IIb transporter protein, expressed in alveolar type II cells leads to the formation of extensive pulmonary intra-alveolar microliths [2]. PAM is often discovered incidentally by chest radiographs obtained for other purposes during early adulthood [1]. The disease presentation is distinctively characterized by mild clinical symptoms at time of diagnosis, and an out of proportion pathognomonic radiographic lung parenchymal involvement. PAM is rarely associated with extrapulmonary or cardiac involvement [3].

Herein, we report the case of a 45-year-old Jordanian man with extensive familial PAM and tricuspid valve calcification.

This case study was performed and is being reported in line with the CARE criteria.

## 2 Presentation of case

**Fig. 1:**
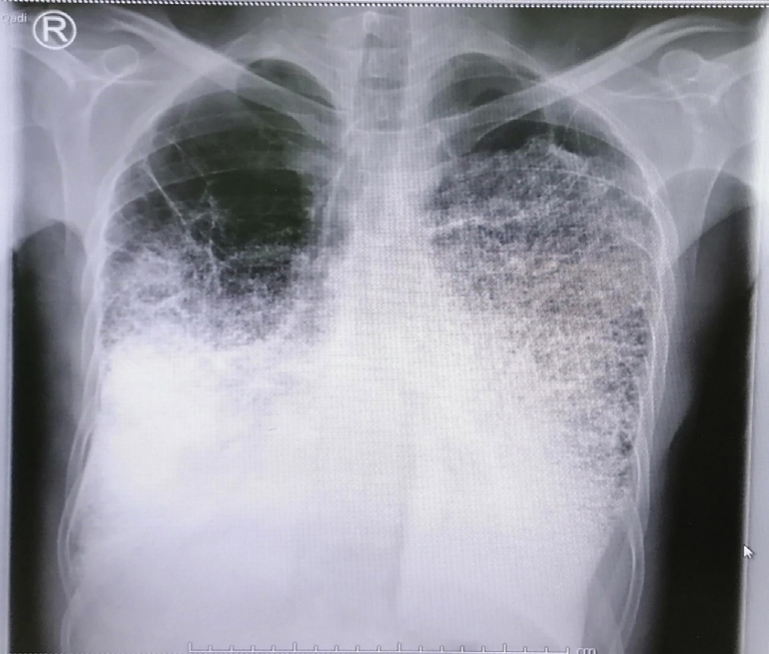
Chest x-ray with bilateral reticulonodular shadowing.

**Fig. 2:**
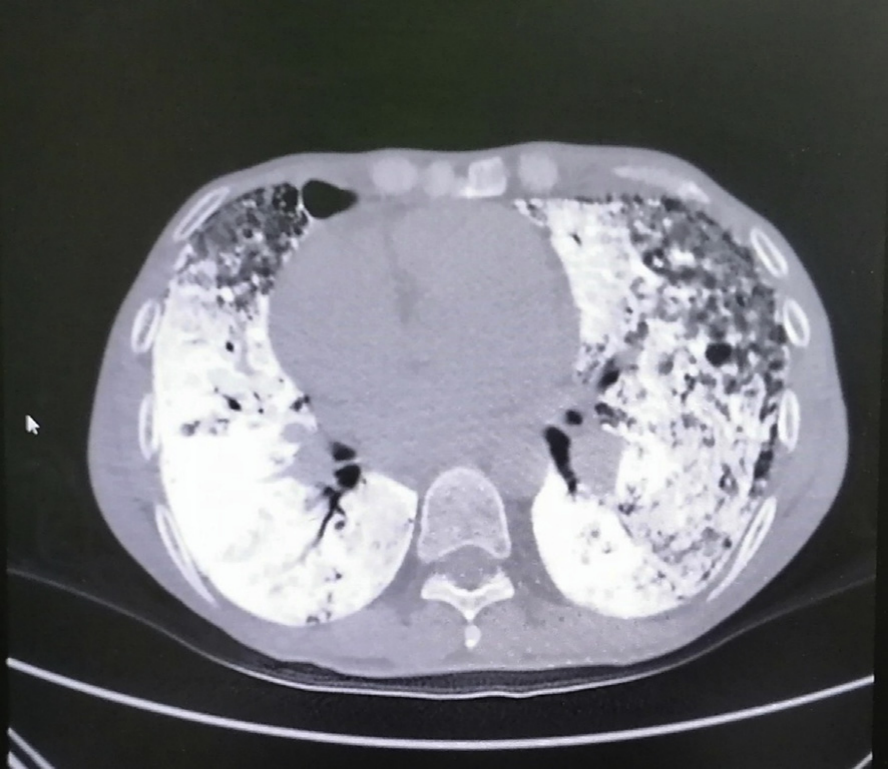
High-resolution CT scan of the chest showing the classical crazy-paving appearance of pulmonary alveolar microlithiasis.

**Fig. 3:**
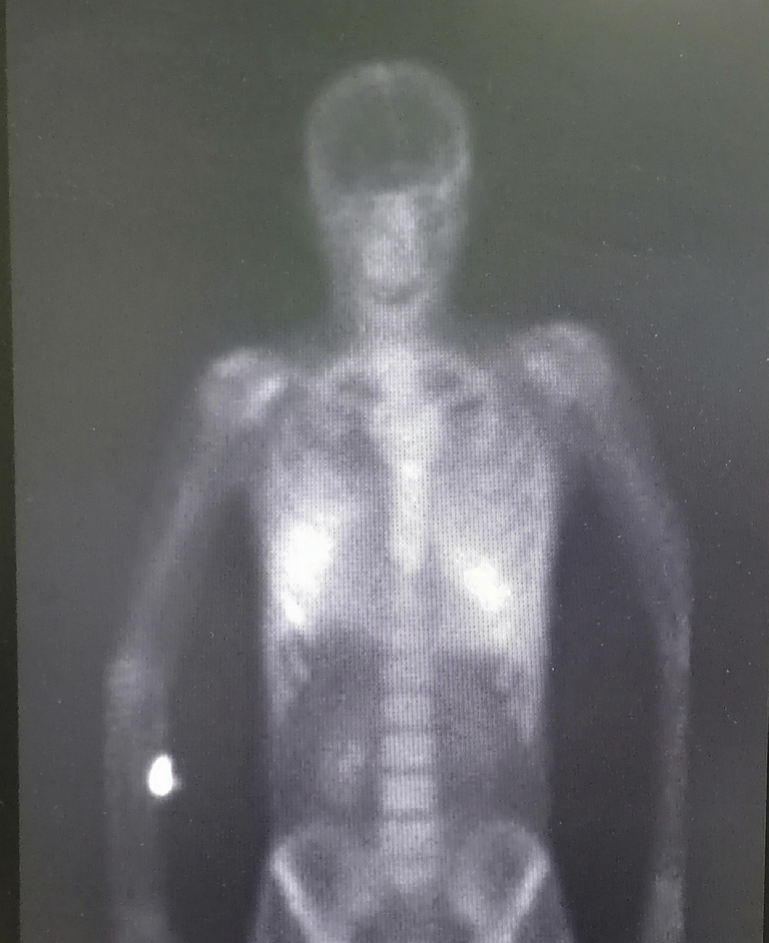
Tc-99 m HD whole body bone scan revealed diffuse increased radiotracer activity in the mid and lower zones of both lungs.

A 45-year-old male patient presented to our center with progressive shortness of breath and dry cough. He had a history of frequent admissions to other hospitals with similar complaints over the past 3 months. He denied any history of fever. He was a heavy smoker with more than 90 pack–years history of smoking. He also worked with concrete and asphalt road construction for more than 15 years. There was no history of exposure to organic dust. On physical examination, he was tachypneic and chest examination revealed diminished breath sounds with bilateral early inspiratory crackles. He had no finger clubbing. Arterial blood gases, on room air at rest, showed pH 7.44, PaCO2 55 mmHg, PaO2 49 mmHg, HCO3 35 mmol/L and SpO2 86.6%. He had normal white blood cells counts with hemoglobin of 14.3 g/dl and the rest of his laboratory workup including comprehensive metabolic profile and liver enzymes were within normal range. CRP and ESR were within normal range. Admission chest radiograph showed bilateral calcific reticulonodular shadowing more prominent in the lower and middle zones with emphysematous changes seen in the in bilateral upper lobes (Fig. 1). High resolution CT scan of the chest revealed diffuse alveolar and interlobular septal prominent calcifications with the characteristic “snowstorm” appearance consistent with PAM with superimposed bilateral patchy ground glass opacities with middle to lower zones predominance and sparing the subpleural spaces (Fig. 2). A Tc-99 m HDP whole-body bone scan revealed increased radiotracer activity in the middle and lower zones of both lungs with no evidence of abnormal focal increased uptake elsewhere (Fig. 3). Echocardiography demonstrated calcifications covering the tricuspid valve with elevated right ventricular systolic pressure at 70 mmHg with 2nd degree mitral regurgitation. There was minimal pericardial effusion. Atrial and ventricular dimensions were within normal range with no evidence of diastolic dysfunction, and estimated ejection fraction of 57% (Video 1).

Supplementary video related to this article can be found at https://doi.org/10.1016/j.amsu.2020.05.039

The following is the supplementary data related to this article:

Video 1: Echocardiography indicating the presence of tricuspid valvular calcifications.

Patient recalled a routine chest radiography he had as part of fitness assessment for military enlistment during his early adulthood and was told to have a pattern of reticulonodular abnormality in both lung fields. He never sought medical attention as he had no respiratory complaints except for minimal exertional dyspnea to strenuous and vigorous activities. His parents are close relatives. He had 11 sisters and 2 brothers. He reported having two sisters with similar illnesses and chest radiographic abnormalities, one died at the age of 38 years from respiratory failure and the other was 42-year-old and still alive and was diagnosed with PAM. The patient and his mother, reported at least 35 close relatives known to have PAM.

During his hospitalization, he was given a course of IV systemic steroids and supplemental oxygen therapy in addition to bronchodilators with minimal improvement. After counseling with the patient, he was discharged home on supplemental home oxygen therapy at 2 L/minutes at rest and 4 L/minutes during sleep and exertions and to be referred for lung transplant evaluation. Unfortunately, few days after discharge, he died at home.

## 3 Discussion

PAM is a rare autosomal recessive, genetic disease. Since it was first described almost 150 years ago, about 1200 cases have been reported worldwide. It was first described by Friedrich in 1856, and in 1918, Harbitz provided an accurate radiological description. PAM is characterized by the presence of numerous tiny calculi (calcospherites) and calcifications within the alveolar spaces. The age at time of presentation ranges between 30 and 50 years. Inactivating Mutations of the Solute-Carrier-family-34 (sodium phosphate) member-2 (SCL34A2) gene, which encodes a type IIb sodium phosphate cotransporter, were proved to be responsible for this disease. The mutation leads to reduced phosphate reuptake by type IIb sodium phosphate transporter in the apical membrane of type-II alveolar cells, resulting in intra-alveolar calcium phosphate chelation and microlith formation [1,2].

Sporadic and familial cases of PAM are reported. Male predominance was reported in sporadic cases. Familial cases account for nearly 30–50% of reported cases and is highly associated with consanguinity with no gender predilection seen. Most of the cases are coming from Europe (42.7%) and Asia (40.6%) [2,4]. To the best of our knowledge, this study reports the highest cluster of at least 35 cases of PAM coming from the same family in Jordan, with a high rate of consanguineous marriages reported among them.

Similar to the presentation of our case, in contrast to the radiological findings, which are often severe, the majority of patients with PAM are remarkably asymptomatic at the time of diagnosis and the disease is usually identified incidentally following radiographic examination of the chest for other purposes. This clinico-radiological dissociation is the hallmark of the presentation of PAM. Shortness of breath is the most common symptom in symptomatic patients followed by a dry cough and chest pain. Smoking and infection may accelerate disease progression, but PAM usually has slow course of progression, producing incremental exertional dyspnea with desaturation in young adulthood, and respiratory insufficiency by late middle age. Physical examination may reveal rales and finger clubbing [5].

On chest radiograph, numerous sand-like microliths are seen diffusely scattered, predominantly in the lower two-thirds of the both lungs, obscuring the diaphragmatic, mediastinal, and cardiac borders. The propensity of the disease for the lung bases is probably due to the larger volume and rich blood supply of the lower lobes. Emphysematous bullae may be present in the lung apices, as well as a zone of hyperlucency between the lung parenchyma and the ribs, known as a “black pleural-line” [6]. High resolution CT scan of the chest shows numerous sandstorm-like intra-alveolar calcifications (microliths) throughout the lungs with subpleural and peribronchial distribution, along with ground-glass opacities. The classic “Sandstorm” appearance with predominance of the middle and even more in the lower lung areas, is the typical radiological presentation of PAM [7]. Differential diagnosis of diffuse pulmonary calcifications on CT or chest radiograph includes healed varicella pneumonia, miliary tuberculosis, Aspirated or extravasated contrast material, hyperparathyroidism, pulmonary amyloidosis, hypervitaminosis D, Paget disease, metastatic calcifications associated with chronic renal failure, and occupational lung disease such as silicosis [1,2]. Bone scintigraphy using technetium-99 m labeled diphosphonate compounds have affinity for calcification foci at soft tissue and can detect early pulmonary calcification [8]. Although histopathological diagnosis is done by either open lung or transbronchial biopsy showing intra-alveolar laminated calcium phosphate concretions [2], many authors argue that typical radiological pattern is considered pathognomonic for PAM and precludes the need for a lung biopsy in most cases. In our case, PAM diagnosis was based on the clinical and characteristic radiographic presentation as our patient refused to undergo tissue biopsy to confirm diagnosis.

SLC34A2 is chiefly expressed in alveolar type-II cells, but is also expressed in other epithelial tissues, including mammary glands, the small intestine, kidneys, pancreas, ovaries, liver, testes, placenta, and prostate. Rare extrapulmonary calcifications can result in testicular atrophy, obstructive azoospermia, medullary nephrocalcinosis, nephrolithiasis and cholelithiasis [2]. Microlith deposits outside the lungs are thought to be associated with the penetrance of mutations of the SLC34A2 gene [9,10]. Cardiac involvement has been described in few cases of PAM. Pericardial calcification [11,12], pulmonary hypertension and cor-pulmonale have been described [13,14]. Four cases with mitral stenosis were reported in the literature, of whom one also had aortic stenosis [15,16]. Although type-II sodium/phosphate co-transporter has been recently identified in a human aortic valve [17], concomitant aortic valvular calcification was reported in only 2 cases. One of the cases had an advanced PAM in otherwise healthy individual, in addition to cholelithiasis and idiopathic medullary nephrocalcinosis [18]. The other case was in a renal transplant recipient with PAM, who was described to have mitral and aortic valve calcifications [19]. To the best of our knowledge, our case is the first case of PAM with tricuspid valvular calcification. Furthermore, in contrast to previously reported cases of PAM with valvular involvement, transthoracic echocardiogram in our case has shown calcification deposit involving the tricuspid valve with concomitant regurgitation rather than stenosis.

The overall prognosis of PAM is poor with slow progression to eventually respiratory failure and cor-pulmonale. Death typically occurs 10–15 years after diagnosis [5]. There is no known effective therapy and treatment remains mainly supportive. Various therapies have been tried including therapeutic bronchoscopic lavage, systemic and inhaled steroids, hydroxychloroquine, and bisphosphonate (disodium etidronate) without benefits in slowing down the progressive course of the disease. For patients with end-stage PAM disease, lung transplantation is the only definitive treatment [20]. After PAM is diagnosed in a given patient, family members should be screened by chest radiography, and parents should be counseled that future children are also at risk of developing the disease.

## 4 Conclusion

PAM is a rare hereditary parenchymal lung disease that is prevalent among families with a high rate of consanguinity. It is a fatal, slowly progressive disease characterized by clinical-radiological dissociation. Extrapulmonary calcifications with valvular heart involvement is very rare. This could be due to a variability in the penetrance of SLC34IIb mutation. Further genetic investigation among families with PAM may aide in early detection of the disease and better understanding of the disease genetic characteristics and pathogenesis for future development of a specific targeted genetic therapy.

## Author contribution

All authors contributed significantly and in agreement with the content of the article. All authors presented substantial contributions to the article and participated of correction and final approval of the version to be submitted.

## Registration of research studies

This is a case report.

## Guarantor

Dr Shaher Samrah.

## Consent for publication

The patient's family gave their consent for publication of this Case report and any accompanying images. None of the images contains any patient's identifiers.

## Funding

**No** funding

## Ethical **approval**

Institutional approval was not required for case reports.

## Declaration of competing interest

The authors declare that they have no competing interests.
