# Supplementary material for: Tricuspid valve calcification in familial pulmonary alveolar microlithiasis: A case report
Source: Ann Med Surg (Lond). 2020 May 30;55:256–9. doi: 10.1016/j.amsu.2020.05.039 (PMC7281303; doi:10.1016/j.amsu.2020.05.039)
Supplement: Multimedia component 2 [file mmc2.docx]

CARE Checklist – 2016: Information for writing a case report

**Topic Item Checklist item description Line/Page**

| **Title** | **1** | The words “case report” should be in the title along with the area of focus | __1_____ |
| --- | --- | --- | --- |
| **Key Words** | **2** | Four to seven key words—include “case report” as one of the key words | __1_____ |
| **Abstract** | **3a 3b 3c** | Background: What does this case report add to the medical literature? Case summary: chief complaint, diagnoses, interventions, and outcomes Conclusion: What is the main “take-away” lesson from this case? | __1___  _1__  __1____ |
| **Introduction** | **4** | The current standard of care and contributions of this case—with references (1-2 paragraphs) | 2_______ |
| **Timeline** | **5** | Information from this case report organized into a timeline (table or figure) | 2_______ |
| **Patient Information** | **6a 6b 6c** | De-identified demographic and other patient or client specific information Chief complaint—what prompted this visit?  Relevant history including past interventions and outcomes | 2____  2____  2___ |
| **Physical Exam** | **7** | Relevant physical examination findings | 2+3 ____ |
| **Diagnostic** | **8a** | Evaluations such as surveys, laboratory testing, imaging, etc. | 3_______ |
| **Assessment** | **8b 8c 8d** | Diagnostic reasoning including other diagnoses considered and challenges Consider tables or figures linking assessment, diagnoses and interventions Prognostic characteristics where applicable | 3___  3_____  3___ |
| **Interventions** | **9a 9b 9c 9d** | Types such as life-style recommendations, treatments, medications, surgery Intervention administration such as dosage, frequency and duration  Note changes in intervention with explanation Other concurrent interventions | _3+4_  __3+4_  __3+4__  __3+4 |
| **Follow-up and** | **10a** | Clinician assessment (and patient or client assessed outcomes when appropriate) | 3+4 |
| **Outcomes** | **10b 10c** | Important follow-up diagnostic evaluations  Assessment of intervention adherence and tolerability, including adverse events | 3+4  3+4 |
| **Discussion** | **11a 11b 11c 11d** | Strengths and limitations in your approach to this case  Specify how this case report informs practice or Clinical Practice Guidelines (CPG) How does this case report suggest a testable hypothesis?  Conclusions and rationale | 4-7  4-7  4-7  4-7 |
| **Patient Perspective** | **12** | When appropriate include the assessment of the patient or client on this episode of care | ___4____ |
| **Informed Consent** | **13** | Informed consent from the person who is the subject of this case report is required by most journals | __8___ |
| **Additional Information** | **14** | Acknowledgement section; Competing Interests; IRB approval when required | ____8___ |

[www.care-statement.org](http://www.care-statement.org/) January 31, 2016
